# Pathogeneses of Tobacco and Nicotine.—Neuroses Produced by Tobacco

**Published:** 1873-03

**Authors:** 


### ARTICLE V.

### *Pathogeneses of Tobacco and Nicotine.—Neuroses Produced by Tobacco.*

*Anœmia, Gaitralgia, Vomiting, Hœmatemesis, Neurosis.* —Mr. C.; October 5, 1856. Face pale, sallow, or rather yellow, recalling at first sight the face of the cancerous patient; physiognomy austere, sad, expression indifferent to everything, arising from extreme discouragement; the eye was dull lusterless, as if drawn towards the back of the orbit; the lips were colorless, the mouth dry; the tongue yellow, without any coat; no thirst; no change of the breath or taste; the patient had appetite but could not eat; every meal, however slight, gives place two or three hours after to atrocious pains of the stomach, soon followed by vomiting. Walking, riding, and, above all, the jarring of the rail-car, when the patient is obliged to travel, induce the same symptoms. He said it was just like sea-sickness. It was the same vertiginous nausea, returning in paroxysms, during which the whole body was covered with a cold sweat. Most frequently the vomiting relieved. Sometimes they took place in the morning before breakfast. What he then threw up was a watery fluid, sometimes insipid, at others sour. In two or three attacks at last, he vomited blood in considerable quantities. There was habitual constipation, worst when the patient felt the worst; he never had diarrhœa. All the abdomen, but more especially the epigastrium and hepatic region was so sensitive that the pressure caused by his uniform was intolerable. The pulse presented nothing abnormal; great heat as well as great cold, and, above all, stormy weather exasperated all the symptoms, which sometimes, on the contrary, were relieved spontaneously, and for a longer or shorter course of time, under the influence of other atmospheric conditions, which the patient could hardly state. During these days of respite, which, unhappily, became more and more infrequent, hope revived again at a prospect of health. The pain in the liver and epigastrium did not cease at these times entirely, but it was endurable, and the patient could eat without vomiting. Then, if the wind rose, while, at the same time, the mercury fell in the barometer, the crises become more violent, and nothing would relieve them, unless it were sometimes lying on the back, with hot applications on the stomach, to say nothing, as this must be understood, of an absolute diet for two, three, or more days, though the desire to eat was most craving.

As I had already been informed by the family that an army Surgeon General and several physicians of Versailles had diagnosticated a cancer of the pylorus, or of the duodenum, I carefully examined every region of the abdomen, but discovered no tumor. What, then, was the disease in hand? I declare that, in spite of the very vague name of gastralgia, which I gave it to quiet the patient, I did not know. I prescribed his regimen, however, and ordered staphysagria 12th, as this seemed to correspond to the most striking symptoms. Whether there was a happy coincidence with more propitious weather—which I am quite inclined to believe at present—or whether the staphysagria showed a real, but as it turned out, a temporary efficacy, it seemed at first to produce the most satisfactory results. For fifteen days, at least, my brave captain, who thought himself almost cured, sang the praises of Homœopathy through the whole scale. Then his léave of absence having expired, he went to rejoin his regiment, in garrison at Cherbourg, promising to write to me.

He did write to me, in fact, and only too often. For more than three months I received two letters a week. The raw winds of La Marche were too much for staphysagria, as they were afterwards for arsenic, causticum, coculus, nux vomica, lycopodium, cicuta, sulphur, &c. In fine, the case was desperate.

In January, 1858, breakfasting one day at Versailles, with an elder brother of my ex-patient, for I had not heard from him for eight months, he said to me, as we were about to sit down, "I have reserved a surprise for you, which I am sure will be an agreeable one; you are about to have a companion at table, one raised from the dead." "Your brother!" I cried. "I did not dare speak of him to you." At the same moment the captain entered.

It was, indeed, a great and agreeable surprise; so great that, I confess, I never experienced the like. Let one imagine the personification of health: a full face, jovial and ruddy; a bright eye; a cheerful smile upon the lips; an easy gait; looking ten years younger at least—this is the man whom I knew to be within a step of the grave; this was my former patient, and now my friend, Captain C.

One might be certain that the first words which I uttered were, "Who cured you? Who cured you?" "I cured myself, please," he said gaily, and, whispering in my ear, as if telling me a secret, "I smoked, as, perhaps, you did not know,—[and in fact I did not,]—and one fine morning I stopped smoking, and my cure dates from that day."

Imagine my surprise. How could I suppose that tobacco, of which I used a considerable quantity every day myself, could produce such ravages in the organism. I tried, but in vain, to deprive the captain of this conviction. He was sure of his fact, and my own experience subsequently satisfied me that he was right.—*Dr. Teste—Journal de la Soc. Gall. 2d serie, t. III.*

*Amaurosis Produced by Tobacco.*—In thirty-seven men with idiopathic amaurosis, Hutchinson found twenty-three confessed smokers. He does not hesitate to think that the use of tobacco was concerned in its production, and he always forbids it where there is an incipient amaurosis.

*Amblyopia, Gradual Atrophy of the Opic Nerve.*—A clerk twenty-one years old began to smoke some years ago.

Augmenting his ration two or three pipes aday, he finally smoked a pound or a pound and a-half of English tobacco a day; he gradually lost his sight; he could not read anything smaller than 6 or 7 millimetres. The ophthalmoscope revealed a brillant whiteness of the nerves, indicating atrophy of the white substance.—*The Lancet*, 1863. *Woods worth.*

*Accidents Produced by Tobacco on Children: Anœmia, Chlorosis, Weakness of Intelligence, Hæmoptysis, Phthisis.*—In thirty-eight children (smokers) examined by M. Dequesne, twenty-two had a marked souffle in the carotids, or rather the bruit de diable, palpitations, pallor of countenane, blood poor in globules, difficulty of digestion, sluggish intellects, dullness, apathy, intermittence of pulse, bleeding at the nose, disturbed sleep, superficial ulcerations of the mucous membrane of the mouth, aphthæ. Finally, one case, a boy of thirteen, a desperate smoker since he was seven years old, was taken with hæmoptysis, then had the cough common in cases where there are pulmonary tubercles, and died at the end of eighteen months—at an age when phthysis is commonly rare.—Dequesne, *Gazette de Hœpitaux*, 1861.

But as regards this case of phthisis, produced by smoking tobacco, we find the opposite effect produced by emanations of tobacco, both crude and manufactured.

In fact, Dr. Buef, of Strasburg, considers that the emanations of tobacco, entering the lungs of those manufacturing it, have a salutary effect as far as phthisis is concerned. Six years of observation have confirmed him in this opinion, that phthisis is very rare with such workmen, and that it makes less progress with those who bring the germs of the diseaes with them into the factory.—*Gaz. Med. de Strasbourg, June*, 1842.

Inquiries made in the factories of Bordeaux, Lille and Havre have given the same results. At Havre, where phthisis is frequent, no case has ever been observed in the tobacco factories.—*Ann. d' Hygiene, October*, 1842.

*Agina Pectoris Produced by the Abuse of Tobacco.*—A physician, who quit the use of tobacco on account of the gastric affections which he experienced, felt also when he smoked nocturnal pains coming in paroxysms, and characterized by a contraction of the thorax, with palpitations and neuralgic, darting pains into the neck. He is now quite well.

*Angina Pectoris.*—A man sixty years of age passed the most of the day in smoking. For about a month he has experienced palpitations, with oppression, and pains shooting towards the shoulders. He ceased smoking. The nocturnal attacks disappeared completely, at the same time that the digestive functions improved. At the end of three months he resumed the use of tobacco, and the attacks returned again ; finally, he quit the use of tobacco entirely, and his attacks of angina ceased, to return no more.

*Angina Pectoris.*—A physician fifty years of age, notwithstanding a fine appearance of health, smoked cigarettes as much as his occupation permitted. For sometime past he has had palpitation, with anguish and constriction of the chest occurring in paroxysms either by day or by night. He ceased smoking, and the attacks disappeared. One day he was in company with some smokers, without smoking himself, but he could not help inhaling an atmosphere loaded with tobacco-smoke ; that night he had an attack.

*Angina Pectoris.*—A physician thirty-five years of age, practicing in the country, smoked cigarettes continually in making his visits. For a long time he ate very little, and without appetite. One morning, fasting and smoking, while going to see his patients, he was suddenly seized with anguish about the heart, with traversal constriction in the upper part of the chest ; he could neither walk nor speak ; his pulse was impreceptible ; hands cold ; and the attack lasted half an hour. The patient came to Paris. He quit tobacco by my advice, and returned home, promising to write to me if he had another attack. I have not heard from him since.

*Angina Pectoris.*—A young Spaniard, thirty years old, smoked cigarettes continually; his appetite was nil, his digestion difficult. One evening, when smoking, he was suddenly taken with a violent pain in his chest, as if he had been compressed in a vice. The pulse was impreceptible; the attack lasted ten minutes, and, frightened, he consented to smoke much less. The symptoms of angina did not return.

*Angina Pectoris and Death.*—An old man, seventy-five years of age, hearty and vigorous, smoked a great deal to relieve himself of trouble, notwithstanding some transient attacks of suffocation. On Saturday he had an attack of angina, which lasted about half an hour. The next day he had another. Sunday he was found dead in his bed.

*Applexy.*—Lanzini relates the case of a soldier who had acquired such a habit of using tobacco that he consumed 3 oz. per day. At the age of twirty-two he began to be attacked with vertigo, which was soon followed by a violent apoplexy, which carried him off.—*Journal d' Allemgue. Translated by G. E. S. for Medical Investigator.*

---

